# Necessities for Labor Cases

**Published:** 1911-11

**Authors:** 


### ABSTRACTS

### Necessities for Labor Cases

1. List of articles to be carried in obstetric bag:
Soap—P. D. & Co's. Germicidal Soap.
New hand scrub brush—5c kind.
Bottle bichloride tablets.
Braided Chinese silk for cord.

Pocket case, needles and sutures.

Needle holder.

Fluid extract ergot and 1 ampoule ergotin for hypo. use.

Hypodermic syringe.

Absorbent cotton.

Ten per cent. sol. cocaine.

Forceps.

Small faradic battery, while not absolutely necessary, is often invaluable in cases of suspended animation.

Chloral in solution 10 grs. to teaspoonful.

Half dozen five-grain quinine capsules.

One gown and one pair rubber gloves.

Half ounce bottle ten per cent. solution silver nitrate.

One tube catgut.

2. List of articles to be provided by expectant mother:

Towels.

Chloroform, 4 oz.

Brandy, 2 oz.

Vinegar, 4 oz.

1 bottle antiseptic tablets.

Cake P. D. germicidal soap.

1 roll narrow tape.

1 new fountain syringe.

1 bed pan.

Six to nine dozen sterilized sanitary napkins, same as sold for traveler's use.

1 lb. absorbent cotton.

1 talcum powder can filled with mixture of 1 part salicylic acid and 4 of starch for drying cord.

Castile soap.

8 oz. tinct. green soap.

Dr. S. Myers, Vicksburg., *Miss., Med. Mo.* Oct., 1911.

